# *In vitro* activity of ceftazidime–avibactam and comparators against OXA-48-like Enterobacterales collected between 2016 and 2020

**DOI:** 10.1128/spectrum.01473-23

**Published:** 2024-02-08

**Authors:** Gregory Stone, Mark Wise, Eric Utt

**Affiliations:** 1 Pfizer Inc., Groton, Connecticut, USA; 2 IHMA, Schaumburg, Illinois, USA; Petrified Bugs LLC, Miami, Florida, USA

**Keywords:** antimicrobial susceptibility, ATLAS, ceftazidime–avibactam, Enterobacterales, OXA-48, surveillance

## Abstract

Oxacillinases (OXA)-48-like β-lactamases are one of the most common resistance determinants among carbapenem-resistant Enterobacterales reported globally. Moreover, there is no standard treatment available against organisms producing OXA-48-like enzymes, and they are sometimes difficult to detect, making treatment challenging. The objective of this study was to evaluate the distribution and antimicrobial susceptibility of *bla*_OXA-48-like_ Enterobacterales isolates against ceftazidime–avibactam (CAZ-AVI) and a panel of comparators collected worldwide from 2016 to 2020 as a part of the Antimicrobial Testing Leadership and Surveillance program. Among all the Enterobacterales isolates collected, 1.8% (1,690/94,052) carried *bla*_OXA-48-like,_ and a majority of those were identified as *K. pneumoniae* (86.5%, 1,462/1,690). Among all the *bla*_OXA-48-like_ isolates, 88.9% (1,502/1,690) were extended-spectrum β-lactamase (ESBL)-positive, 20.7% (350/1,690) were metallo-β-lactamase (MBL)-positive, and 8.9% (150/1,690) were ESBL- and MBL-negative. There were 10 different variants of the OXA-48-like family of enzymes detected, with the major variant being *bla*_OXA-48_ (50.2%, 848/1,690), *bla*_OXA-232_ (29.3%, 496/1,690), and *bla*_OXA-181_ (18.0%, 304/1,690). Overall, all the *bla*_OXA-48-like_ isolates showed a susceptibility of 78.6% to CAZ-AVI. Importantly, high susceptibility to CAZ-AVI was shown by all the *bla*_OXA-48_ type, MBL-negative isolates (*n* = 1,380, ≥99.0%), and all the MBL-negative isolates (*n* = 1,300, ≥97.6%) of the major variants (*bla*_OXA-48_*, bla*_OXA-232_, and *bla*_OXA-181_) studied. Among the comparator agents, all isolates showed good susceptibility to only tigecycline (>95.0%) and colistin (>78.6%). Considering the limited treatment options available, CAZ-AVI could be considered as a potential treatment option against *bla*_OXA-48-like_ Enterobacterales. However, routine surveillance and appropriate stewardship strategies for these organisms may help identify emerging resistance mechanisms and effective treatment of infections.

**IMPORTANCE:**

Resistance to carbapenems among Enterobacterales is often due to the production of enzymes that are members of the oxacillinases (OXA)-48-like family. These organisms can also be resistant to other classes of drugs and are difficult to identify and treat. This study evaluated the activity of the drug ceftazidime–avibactam (CAZ-AVI) and other comparator agents against a global collection of Enterobacterales that produce OXA-48-like enzymes. CAZ-AVI was active against *bla*_OXA-48-like_ Enterobacterales, and only colistin and tigecycline were similarly active among the comparator agents, highlighting the limited treatment options against these organisms. Continued surveillance of the distribution of these OXA 48-like producing Enterobacterales and monitoring of resistance patterns along with the implementation of antimicrobial stewardship measures to guide antibiotic use and appropriate treatment are necessary to avoid drug resistance among these organisms.

## INTRODUCTION

Carbapenems are a class of broad-spectrum β-lactam antibiotics, initially approved in 1985 (1). These have been used to treat multidrug-resistant bacteria, including Enterobacterales producing extended-spectrum β-lactamases (ESBLs) and ampicillinase-C (1–3). However, overuse of carbapenems led to the emergence of carbapenem-resistant Enterobacterales (CREs), which was first identified in 1996 (1, 3). Notably, prior to 2001, about 99.9% of Enterobacterales were susceptible to carbapenems (4). CREs have been disseminated worldwide with varying degrees of prevalence (5), with an increase in infection rates of CREs in Europe (1.3/10,000), the United States (2.93/100,000), and China (4/10,000) between 2012 and 2015 (4, 6, 7). Furthermore, a recent global study reported an increase in the prevalence of carbapenem non-susceptible Enterobacterales from 3.3% globally between 2012 and 2017 to 5.7% between 2018 and 2019 (8). Infections caused by CREs have been highlighted as a global health threat, with the Wolrd Health Organization (WHO) categorizing them as critical (highest priority) pathogens (9, 10).

Resistance to carbapenems can be mediated by production of carbapenemases belonging to Ambler class A (*bla*_KPC_ and *bla*_GES_), class B [metallo-β-lactamases (MBL)], or class D [oxacillinases (OXA)] β-lactamases (10, 11). Among the resistance determinants of CREs, those carrying metallo-β-lactamases (MBLs, 36.7%), *bla*_KPC_ (25.5%), and *bla*_OXA-48-like_ (24.1%) have been shown to be most frequently identified as per a global study assessing distribution between 2018 and 2019 (8). Notably, the resistance mechanisms among CREs vary among the different regions, with predominance of *bla*_NDM_ reported in Africa and the Middle East (AfME) and Asia Pacific (APAC), *bla*_OXA-48-like_ in Europe, and *bla*_KPC_ in Latin America (LATAM) and North America (8). Furthermore, CRE infections are associated with an increased risk of morbidity and mortality and higher cost (9, 12–14). Two studies assessing the cost associated with CRE infections reported a higher cost: an incidence of 15 per 100,000 costing USD 1.4 billion to the hospitals, USD 0.8 billion to third-party payers, and USD 2.8 billion in the United States (12); and a hospital-associated CRE incidence of 233 per 100,000 costed SGD 12.16 million annually based on direct costs in Singapore (13).

OXA, belonging to class D β-lactamases, are either plasmid-mediated or naturally occurring chromosomally encoded (15). There are several groups of carbapenem-resistant OXA-type β-lactamases, including OXA-23-like, OXA-24/40-like, OXA-51-like, OXA-58-like, and OXA-48-like (16). The OXA-48 enzyme was first identified in *Klebsiella pneumoniae* in Turkey in 2001 (17). Following that, they were also reported in multiple countries in the Middle East and North Africa, which were important reservoirs for OXA-48-like Enterobacterales (18). Since then, they have been reported as the most common carbapenemases in several regions spreading to non-endemic areas, causing nosocomial outbreaks (19–21). There are currently no standard treatments against infections caused by isolates producing these carbapenemases (21, 22). Furthermore, currently, these bacteria are sometimes difficult to detect due to their lower carbapenem-hydrolyzing ability as compared to other carbapenemases, and as a result, they often do not exceed the level for detection of phenotypic resistance. This delay in detection could lead to treatment errors and is possibly associated with treatment failure (21).

Although extended-spectrum cephalosporins are active against OXA-48-like enzymes, most OXA-48-like enzymes containing bacteria also carry ESBLs, making them resistant to treatment with these cephalosporins (21). Ceftazidime–avibactam (CAZ-AVI) is a combination of ceftazidime, a third-generation cephalosporin and avibactam, and a non-β-lactam β-lactamase inhibitor (23). CAZ-AVI has been shown to be active against *bla*_OXA-48-like_ Enterobacterales, including those carrying ESBLs (20, 21, 24). However, CAZ-AVI is not active against organisms that produce MBLs (25). This activity of CAZ-AVI is attributed to the activity of avibactam against ambler class A, class C, and some class D β-lactamases but not class B (26). The addition of avibactam to ceftazidime expands the Gram-negative spectrum of activity to include multidrug-resistant (MDR) bacteria, those producing ESBLs and non-MBLs (26).

Due to the increasing incidence, limited treatment options, and challenges in detection of Enterobacterales producing OXA-48-like carbapenemases, it is important to continue monitoring the distribution and resistance patterns of these isolates to establish appropriate antibiotic stewardship strategies for the management of infections caused by these organisms. A previous study, which was a part of the International Network for Optimal Resistance Monitoring (INFORM) global surveillance program, reported the distribution and susceptibility of *bla*_OXA-48-like_ Enterobacterales isolates to CAZ-AVI, collected between 2012 and 2015 from Africa and the Middle East (AfME), Asia Pacific (APAC), Europe, and Latin America (LATAM). The study reported that CAZ-AVI demonstrated good activity (susceptibility 89.7%, MIC_90_ >128 mg/L) against *bla*_OXA-48-like_ isolates, with potent activity (susceptibility 100%, MIC_90_ 4 mg/L) against the MBL-negative isolates (25). The current study provides an update to the previous study and aims to evaluate the distribution and antimicrobial susceptibility of *bla*_OXA-48-like_ Enterobacterales isolates collected worldwide (AfME, APAC, Europe, LATAM, and North America) in 2016–2020 from the Antimicrobial Testing Leadership and Surveillance (ATLAS) program (27) against CAZ-AVI and a panel of comparator agents.

## RESULTS

**Fig 1:**
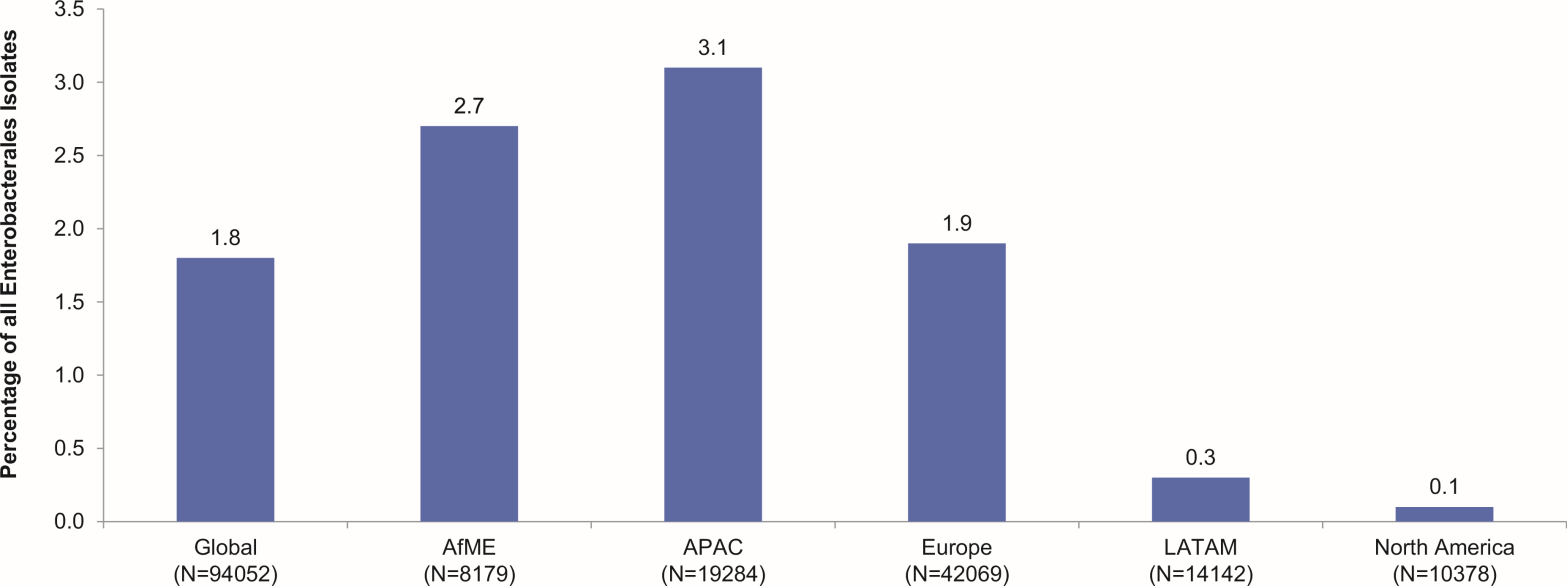
Distribution of *bla*_OXA-48-like_ Enterobacterales isolates collected globally and across different regions in 2016–2020. N, total number of Enterobacterales collected; n, number of *bla*_OXA-48-like_ isolates. The number of *bla*_OXA-48-like_ Enterobacterales identified globally among all Enterobacterales isolates collected was 1690. These isolates were identified from AfME (*n* = 220), APAC (*n* = 598), Europe (*n* = 812), LATAM (*n* = 49), and North America (*n* = 11).

A total of 94,052 Enterobacterales isolates were collected from 320 sites in 60 countries between 2016 and 2020 (Fig. 1; Table S2). Most of the isolates were identified as *Escherichia coli*, 29,274 (31.1%) and *Klebsiella* pneumoniae, 26,510 (28.2%; Table 1).

**TABLE 1:** Distribution of *bla*_OXA-48-like_-carrying isolates among different organisms of Enterobacterales collected globally in 2016–2020^*e*^

Organisms [*n* (% of N)]	All organisms (*N* = 94,052)	OXA-48-like (*N* = 1,690)
*Escherichia coli*	29,274 (31.1%)	107 (6.3%)
*Klebsiella pneumoniae*	26,510 (28.2%)	1,462 (86.5%)
*Enterobacter cloacae*	7,117 (7.6%)	43 (2.5%)
*Citrobacter* spp.	5,879 (6.3%)	17 (1.0%)
*Enterobacter* spp.^*a*^	1,002 (1.1%)	1 (0.1%)
*Klebsiella* spp.^*b*^	8,899 (9.5%)	17 (1.0%)
*Proteus* spp.	6,138 (6.5%)	5 (0.3%)
*Serratia* spp.	4,030 (4.3%)	9 (0.5%)
*Morganella morganii*	2,865 (3.0%)	1 (0.1%)
*Providencia* spp.	2,139 (2.3%)	27 (1.6%)
Others^*c*^^,*d*^	145 (0.2%)	1 (0.1%)

^*a*^ Excludes isolates of *E. cloacae.*

^*b*^ Excludes isolates of *K. pneumoniae.*

^*c*^ Other organisms among all organisms include *Raoultella* spp. (*n* = 117), *Pluralibacter gergoviae* (*n* = 12), *Pantoea* spp. (*n* = 9), *Cronobacter sakazakii* (*n* = 2), *Lelliottia amnigena* (*n* = 2), *Escherichia vulneris* (*n* = 1), *Hafnia alvei* (*n* = 1), and *Kluyvera ascorbate* (*n* = 1).

^*d*^ Other organisms among OXA-48‑like Enterobacterales include *Raoultella* spp. (*n* = 1).

^*e*^ N, total number of isolates; n, number of isolates of each organism.

**Fig 2:**
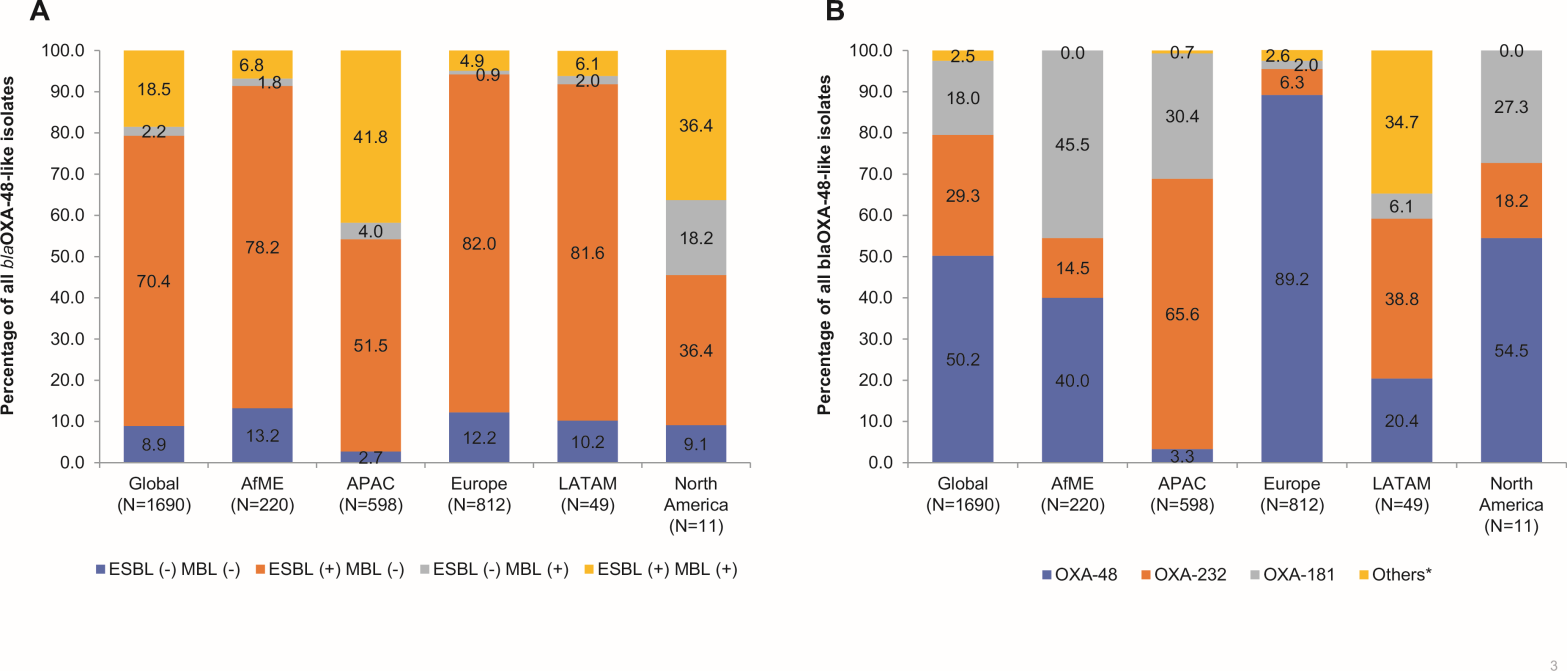
Distribution of *bla*_OXA-48-like_, ESBL, and *bla*_OXA-48-like_ variants among Enterobacterales collected globally and across different regions in 2016–2020. AfME, Africa and Middle East; APAC, Asia Pacific; ESBL, extended-spectrum β-lactamase LATAM, Latin America; MBL, metallo-β-lactamase; N, total number of *bla*_OXA-48-like_ Enterobacterales collected. (**A**) Distribution of *bla*_OXA-48-like_, ESBL Enterobacterales isolates collected globally and across different regions in 2016–2020. The number of isolates collected globally was ESBL (−), MBL (−), *n* = 150 (AfME, *n* = 29; APAC, *n* = 16; Europe, *n* = 99; LATAM, *n* = 5; North America *n* = 1); ESBL (+), MBL (−), *n* = 1190 (AfME, *n* = 172; APAC, *n* = 308; Europe, *n* = 666; LATAM, *n* = 40; North America *n* = 4); ESBL (−), MBL (+), *n* = 38 (AfME, *n* = 4; APAC, *n* = 24; Europe, *n* = 7; LATAM, *n* = 1; North America *n* = 2); ESBL (+), MBL (+), *n* = 312 (AfME, *n* = 15; APAC, *n* = 250; Europe, *n* = 40; LATAM, *n* = 3; North America *n* = 4). (**B**) Distribution of *bla*_OXA-48-like_ variants among Enterobacterales collected globally and across different regions in 2016–2020. Total number of isolates of variants collected included the following: OXA-48, *n* = 848 (AfME, *n* = 88; APAC, *n* = 20; Europe, *n* = 724; LATAM, *n* = 10; North America, *n* = 6); OXA-232, *n* = 496 (AfME, *n* = 32; APAC, *n* = 392; Europe, *n* = 51; LATAM, *n* = 19; North America, *n* = 2); OXA-181, *n* = 304 (AfME, *n* = 100; APAC, *n* = 182; Europe, *n* = 16; LATAM, *n* = 3; North America, *n* = 3); others, *n* = 42. *Others include OXA-244 (*n* = 17, *n* = 1 in APAC, and *n* = 16 in Europe), OXA-163 (*n* = 12 in LATAM), OXA-162 (*n* = 5 in Europe), OXA-370 (*n* = 5 in LATAM), OXA-484 (*n* = 1 in APAC), OXA-48-TYPE (*n* = 1 in APAC), and OXA-48-new variant (*n* = 1 in APAC).

Globally, 1.8% (1,690/94,052) of all Enterobacterales were *bla*_OXA-48-like_ (Fig. 1; Table S2). The majority of *bla*_OXA-48-like_ isolates were identified as *K. pneumoniae* (86.5%, 1,462/1,690; Table 1). Among the different geographical regions, Enterobacterales isolates collected in APAC had the highest proportion of *bla*_OXA-48-like_ β-lactamases (3.1%, 598/19,284), and those collected in North America had the lowest (0.1%, 11/10,378; Fig. 1; Table S2). Among all the *bla*_OXA-48-like_ Enterobacterales isolates, 88.9% (1,502/1,690) were ESBL-positive, 20.7% (350/1,690) were MBL-positive, and 8.9% (150/1,690) were ESBL- and MBL-negative (Fig. 2A; Table S2). There were 10 different variants of the OXA-48-like family of enzymes detected among the Enterobacterales isolates. Among these, the highest proportion carried *bla*_OXA-48_ (50.2%, 848/1,690), followed by *bla*_OXA-32_ (29.3%, 496/1,690) and *bla*_OXA-181_ (18.0%, 304/1,690). Among the different geographical regions, the proportion of *bla*_OXA-48_ proper was the highest among isolates collected in Europe (89.2%, 724/812), followed by North America (54.5%, 6/11); a similar proportion of isolates in AfME carried *bla*_OXA-48_ (40.0%, 88/220) and *bla*_OXA-181_ (45.5%, 100/220), and *bla*_OXA-232_ was the most common variant among *bla*_OXA-48-like_ isolates in APAC (65.6%, 392/598) and LATAM (38.8%, 19/49; Fig. 2B; Table S3).

Antibiotic susceptibility for CAZ-AVI against all *bla*_OXA-48-like_ isolates was 78.6% with an MIC_90_ of >128 mg/L (Table 2). Importantly, CAZ-AVI showed high antimicrobial activity against the MBL-negative isolates, including OXA-48-like, MBL (−), ESBL (±) (susceptibility, 99.0%; MIC_90_, 2 mg/L) and OX-48-like, ESBL (+), MBL (−) isolates (susceptibility, 99.1%, MIC_90_, 2 mg/L). Among the comparator agents, only tigecycline (susceptibility, 94.0%–96.0%; MIC_90_, 2 mg/L) and colistin (susceptibility, 81.8%–84.0%; MIC_90_, >8 mg/L) were active against all the isolates (Table 3).

**TABLE 2:** Antimicrobial activity of CAZ-AVI and comparators against all *bla*_OXA-48-like_ Enterobacterales isolates collected globally in 2016–2020^*c*^

	%S	%R	MIC_50_	MIC_90_	MIC range
*N* = 1690					
Amikacin	56.1	39.5	8	>64	0.25–>64
Aztreonam	9.5	89.5	>64	>128	0.03–>128
CAZ-AVI	78.6	21.4	1	>128	0.015–>128
Cefepime	6.9	87.9	>32	>32	0.12–>32
Colistin^*a*^	81.9	18.1	0.5	>8	0.06–>8
Imipenem	10.4	76.4	8	>8	0.06–>8
Meropenem	17	71.5	16	>16	0.015–>16
Pip/Taz	0.9	98.9	>64	>128	0.25–>128
Tigecycline^*b*^	95.5	5.3	1	2	0.06–>8

^*a*^ EUCAST breakpoints have been used.

^*b*^ FDA-approved breakpoints have been used.

^*c*^ N, total number of isolates; S, susceptibility; R, resistance; CAZ-AVI, ceftazidime–avibactam; Pip/taz, piperacillin/tazobactam.

**TABLE 3:** Antimicrobial activity of CAZ-AVI and comparators against *bla*_OXA-48-like_. ESBL Enterobacterales isolates collected globally in 2016–2020^*c*^

	%S	%R	MIC_50_	MIC_90_	MIC range
*bla*_OXA-48-Like_, ESBL (−), MBL (−) (*N* = 150)					
Amikacin	76.0	20.0	2	>64	0.5–>64
Aztreonam	75.3	20.1	0.25	32	0.03–128
CAZ-AVI	98.7	1.3	0.5	1	0.015–>128
Cefepime	66.0	7.3	2	8	0.12–>32
Colistin^*a*^	84.0	16.0	0.5	>8	0.06–>8
Imipenem	30.0	44.0	4	>8	0.25–>8
Meropenem	45.3	40.0	4	>16	0.03–>16
Pip/Taz	6.7	99.3	>64	>128	8–>128
Tigecycline^*b*^	94.0	1.3	0.5	2	0.06–8
*bla*_OXA-48-Like_, ESBL (+), MBL (−) (*N* = 1190)					
Amikacin	64.6	30.7	4	>64	0.25–>64
Aztreonam	1.8	97.5	128	>128	0.12–>128
CAZ-AVI	99.1	0.9	1	2	0.015–>128
Cefepime	1.4	94.8	>32	>32	0.12–>32
Colistin^*a*^	81.8	18.2	0.5	>8	0.06–>8
Imipenem	29.4	50.2	8	>8	0.12–>8
Meropenem	22.5	65.5	>8	>16	0.015–>16
Pip/Taz	1.0	98.7	>64	>128	0.25–>128
Tigecycline^*b*^	96.0	0.5	1	2	0.06–>8
*bla*_OXA-48-Like_, ESBL (±), MBL (−) (*N* = 1340)					
Amikacin	65.9	32.3	4	>64	0.25–>64
Aztreonam	10.0	88.9	128	>128	0.03–>128
CAZ-AVI	99.0	1.0	1	2	0.015–>128
Cefepime	8.7	85.0	>32	>32	0.12–>32
Colistin^*a*^	82.0	18.0	0.5	>8	0.06–>8
Imipenem	12.9	70.5	4	>8	0.12–>8
Meropenem	21.3	64.3	>8	>16	0.015–>16
Pip/Taz	1.0	98.7	>64	>128	0.25–>128
Tigecycline^*b*^	95.8	0.6	1	2	0.06–>8

^*a*^ EUCAST breakpoints have been used.

^*b*^ FDA-approved breakpoints have been used.

^*c*^ N, total number of isolates; S, susceptibility; R, resistance; CAZ-AVI, ceftazidime–avibactam; Pip/taz, piperacillin/tazobactam; ESBL, extended-spectrum β-lactamase; MBL, metallo-β-lactamase.

Against the major variants of *bla*_OXA-48-like_ isolates, CAZ-AVI showed good activity against *bla*_OXA-48_ isolates (susceptibility, 91.9%; MIC_90_, 2 mg/L) but lower activity against *bla*_OXA-181_ (susceptibility, 60.2%; MIC_90_, 8 mg/L) and *bla*_OXA-232_ isolates (susceptibility, 65.7%; MIC_90_, >128 mg/L mg/L). However, when the MBL-producing isolates were removed from the data set, CAZ-AVI showed good antimicrobial activity against all three *bla*_OXA-48-like_ variants (susceptibility, 97.6%-99.5%; MIC_90_, 2 mg/L). Among the comparator agents, tigecycline showed good antimicrobial activity against all the variants (susceptibility, 91.9%–97%; MIC_90_, 2 mg/L). Colistin showed good antimicrobial activity against all *bla*_OXA-181_ (susceptibility, 86.8%; MIC_90_, 8 mg/L) and *bla*_OXA-232_ isolates (susceptibility, 84.7%; MIC_90_, 8 mg/L; Table 4).

**TABLE 4:** Antimicrobial activity of CAZ-AVI and comparators against variants of *bla*_OXA-48l-like_ Enterobacterales isolates collected globally in 2016–2020^*c*^

	%S	%R	MIC50	MIC90	MIC range
*bla*_OXA-48_ (*N* = 848)					
Amikacin	76.1	18.0	4	>64	0.25–>64
Aztreonam	13.1	86.0	128	>128	0.03–>128
CAZ-AVI	91.9	8.1	0.5	2	0.015–>128
Cefepime	11.7	81.7	32	>32	0.12–>32
Colistin^*a*^	78.7	21.3	0.25	0.5	0.06–>8
Imipenem	9.0	71.1	4	8	0.12–>8
Meropenem	38.6	49.1	>16	>16	0.06–>16
Pip/Taz	1.1	98.7	>64	>128	0.25–>128
Tigecycline^*b*^	95.6	0.9	0.5	2	0.06–8
*bla*_OXA-48_, MBL (−) (*N* = 783)					
Amikacin	79.2	20.8	4	>64	0.25–>64
Aztreonam	13.2	86.0	128	>128	0.03–>128
CAZ-AVI	99.5	0.5	0.5	2	0.015–>128
Cefepime	12.6	80.3	>16	>32	0.12–>32
Colistin^*a*^	78.9	21.0	0.5	>8	0.06–>8
Imipenem	9.7	68.8	4	>8	0.12–>8
Meropenem	20.7	58.2	2	>16	0.015–>16
Pip/Taz	1.2	98.6	>64	>128	0.25–>128
Tigecycline^*b*^	95.9	0.9	0.5	2	0.06–8
*bla*_OXA-181_ (*N* = 304)					
Amikacin	51.6	46.4	16	>64	0.5–>64
Aztreonam	6.6	91.8	>64	>128	0.03–>128
CAZ-AVI	60.2	39.8	2	>128	0.06–>128
Cefepime	4.0	92.1	>32	>32	0.12–>32
Colistin^*a*^	86.8	13.2	0.25	8	0.06–>8
Imipenem	28.6	62.5	>8	>8	0.12–>8
Meropenem	30.6	67.8	>16	>16	0.06–>16
Pip/Taz	0.3	99.0	>64	>128	2–>128
Tigecycline^*b*^	92.1	0.3	0.5	2	0.06–>8
*bla*_OXA-181_, MBL (−) (*N* = 184)					
Amikacin	78.8	19.0	4	>64	0.5–>64
Aztreonam	7.1	91.3	>64	>128	0.12–>128
CAZ-AVI	99.5	0.5	1	2	0.06–64
Cefepime	6.0	87.5	>32	>32	0.12–>32
Colistin^*a*^	91.3	8.7	0.25	2	0.06–>8
Imipenem	26.1	52.7	4	>8	0.12–>8
Meropenem	39.6	49.5	2	>16	0.06–>16
Pip/Taz	0.0	98.9	>64	>128	16–>128
Tigecycline^*b*^	91.9	0.5	0.5	2	0.06–>8
*bla*_OXA-232_ (*N* = 496)					
Amikacin	23.4	73.4	>64	>64	0.25–>64
Aztreonam	4.6	95.2	>64	>128	0.12–>128
CAZ-AVI	65.7	34.3	1	>128	0.03–>128
Cefepime	0.4	96.6	>32	>32	2–>32
Colistin^*a*^	84.7	15.3	0.5	8	0.12–>8
Imipenem	8.3	81.7	8	≥8	0.06–>8
Meropenem	6.1	92.7	>16	>16	0.25–>16
Pip/Taz	0.4	99.6	>64	>128	2–>128
Tigecycline	97.0	0.0	1	2	4.0–8
*bla*_OXA-232_, MBL (−) (*N* = 333)					
Amikacin	26.4	73.0	>64	>64	1–>64
Aztreonam	3.9	95.8	>64	>128	0.12–>128
CAZ-AVI	97.6	2.4	1	2	0.015–>128
Cefepime	0.6	95.2	>32	>32	2–>32
Colistin^*a*^	84.4	15.6	0.5	8	0.12–>8
Imipenem	9.3	88.3	8	>8	0.5–>8
Meropenem	8.4	89.8	>16	>16	0.25–>16
Pip/Taz	0.3	99.7	>64	>64	1–>128
Tigecycline^*b*^	97.0	0.0	1	2	0.12–10

^*a*^ EUCAST breakpoints have been used.

^*b*^ FDA-approved breakpoints have been used.

^*c*^ N, total number of isolates; S, susceptibility; R, resistance; CAZ-AVI, ceftazidime–avibactam; Pip/taz, piperacillin/tazobactam; ESBL, extended-spectrum β-lactamase; MBL, metallo-β-lactamase.

## DISCUSSION

This study assessed the distribution and antimicrobial susceptibility of *bla*_OXA-48-like_ Enterobacterales isolates against CAZ-AVI and a panel of comparator agents collected globally from 2016 to 2020. Among all the Enterobacterales isolates collected, 1.8% carried *bla*_OXA-48-like_ β-lactamases, with the majority of the isolates (86.5%) identified as *K. pneumoniae.* Majority of the *bla*_OXA-48-like_ isolates co-carried ESBLs (88.9%). Of note, 10 different variants of *bla*_OXA-48-like_ β-lactamases were detected among Enterobacterales, with *bla*_OXA-48_ (50.2%), *bla*_OXA-232_ (29.3%), and *bla*_OXA-181_ (18.0%) being the most common. Overall, *bla*_OXA-48-like_ Enterobacterales showed good susceptibility to CAZ-AVI (78.6%). For MBL-negative *bla*_OXA-48-like_ Enterobacterales, CAZ-AVI showed potent antimicrobial activity (susceptibility, 99.0%; MIC_90_ 2 mg/L). Among the comparator agents, tigecycline and colistin were the only agents that were active against all *bla*_OXA-48-like_ Enterobacterales.

Our study identified 1.8% of all Enterobacterales isolates collected as *bla*_OXA-48-like_. The INFORM study, which included isolates collected between 2012 and 2015, had identified 0.73% of all isolates as *bla*_OXA-48-like_ (25). However, the previous study did not include isolates collected in North America. In the current study, only a small (0.7%, 11/1,690) proportion of the total *bla*_OXA-48-like_ Enterobacterales isolates collected were in North America. Our study revealed that 8.9% of all the *bla*_OXA-48-like_ isolates did not co-carry any other β-lactamases (ESBL-negative; MBL-negative), which is a reduction from the 13.8% reported in the previous INFORM study. Accordingly, there was an increase in the proportion of *bla*_OXA-48-like_ isolates co-carrying ESBLs (2016–2020 vs 2012–2015, 88.9% vs 77.5%) or MBLs (20.7% vs 9.0%) (25).

Among the variants of *bla*_OXA-48-like_ Enterobacterales, a majority of them were *bla*_OXA-48_ (50.2%, 848/1,690). OXA-48 has been reported as the most abundant OXA-48-like enzyme, globally (19). In this study, a majority of the *bla*_OXA-48_ isolates collected globally were from Europe (85.4%, 724/848), followed by AfME (10.4%, 88/848; Fig. 2B; Table S3). Furthermore, Russia (27.6%, 200/724), Turkey (21.2%, 154/724), and Spain (15.3%, 111/724) in Europe and Morocco (42.0%, 37/88) and South Africa (15.9%, 14/88; data not shown) in AfME contributed most of the *bla*_OXA-48_ isolates. Turkey and Morocco have been reported to be endemic for *bla*_OXA-48_-carrying Enterobacterales with subsequent reports in Spain and South Africa after early nosocomial outbreaks, which are in line with the findings of our study (19). Notably, increasing prevalence and spread of *bla*_OXA-48_-carrying Enterobacterales in Russia have been reported in a previous global surveillance study in which the highest number of OXA-48-producing isolates were from Russia in 2018 (28). In the current study, *bla*_OXA-232_ (29.3%, 496/1,690), and *bla*_OXA-181_ (18.0%, 304/1,690) were the other variants frequently identified. However, OXA-181 was previously reported to be more frequent than OXA-232 (19). Furthermore, in the previous INFORM study, while *bla*_OXA-48_ was the most common variant (79.6%), other major variants detected were *bla*_OXA-181_ (7.5%), *bla*_OXA-163_ (3.9%), *bla*_OXA-232_ (3.6%), and *bla*_OXA-244_ (3.6%) (25). A majority of the *bla*_OXA-232_ were collected in APAC (79.0%, 392/496) and Europe (10.3%, 51/496), while those of *bla*_OXA-181_ were collected in APAC (59.9%, 182/304) and AfME (32.9%, 100/304). Both OXA-181 and OXA-232 have been reported to be endemic to India, which supports the findings of the current study in which 69.4% (344/496) of *bla*_OXA-232_ and 59.2% (160/304) of *bla*_OXA-181_ were collected in India (data not shown) (19). Thailand (9.5%, 47/496) and Turkey (7.7%, 38/496) were the other major contributors of *bla*_OXA-232_ isolates in our study (data not shown). Notably, OXA-232 has been reported in Thailand and Europe since 2015 (19). South Africa was the other major contributor of *bla*_OXA-181_ (27.6%, 84/304; data not shown), which has been previously reported (19).

Our study demonstrated high susceptibility (>99.0%, Table 4) to CAZ-AVI against all the *bla*_OXA-48-like_, MBL (−), ESBL (±) and the ESBL (+), MBL (−) isolates, which was similar to the susceptibility (>99.2%) among those collected in 2012–2015 (25). In the current study, CAZ-AVI was active against all isolates of *bla*_OXA-48-like_ Enterobacterales (78.6%, Table 4). Comparatively higher susceptibility to CAZ-AVI was reported in the INFORM study (89.3%–92.5%) (25). In the current study, CAZ-AVI showed very low activity (susceptibility, ≤0.3%; MIC_90_, >128 mg/L; Table S2) against *bla*_OXA-48-like_, MBL (+), ESBL (±) Enterobacterales isolates, which is expected as avibactam has no inhibitory activity against MBLs (24). However, to overcome the lack of activity of avibactam against MBL-positive Enterobacterales, the addition of aztreonam to CAZ-AVI has been proposed as a therapeutic combination (29). In line with our current finding that CAZ-AVI was active against MBL-negative isolates, the overall reduced susceptibility of all *bla*_OXA-48-like_ Enterobacterales isolates to CAZ-AVI, as compared to the previous INFORM study, could be attributed to the increase in the proportion of *bla*_OXA-48-like_ isolates co-carrying MBLs in the current study (25). Taken together, while CAZ-AVI still presents a potential treatment option against these isolates, the rise in the proportion of isolates co-carrying MBLs that trigger CAZ-AVI resistance is of serious concern.

Among the major *bla*_OXA-48-like_ variants, all isolates-carrying *bla*_OXA-48_ showed high susceptibility (91.9%) to CAZ-AVI as compared to those carrying *bla*_OXA-232_ (65.7%) and *bla*_OXA-181_ (60.2%). In the previous INFORM study, the overall susceptibility of *bla*_OXA-48 type_ isolates to CAZ-AVI (92.5%) was in line with that reported in this study. However, all isolates of *bla*_OXA-181_ and 41.7% of *bla*_OXA-232_ were susceptible to CAZ-AVI in the previous study (25). Importantly, MBL-negative isolates of all the three major variants assessed in the current study showed high susceptibility (>97.6%) to CAZ-AVI. These data are corroborated by the previous INFORM study in which MBL-negative isolates of the three *bla*_OXA-48-like_ variants showed high susceptibility (>99.2%) to CAZ-AVI. Notably, MBL co-carriage was high among *bla*_OXA-232_ (32.9%)- and *bla*_OXA-181_ (39.5%)-carrying isolates, indicating that the lower susceptibility to CAZ-AVI among these variants is likely due to the presence of MBL genes (Table S3).

Among the comparator agents, only tigecycline (susceptibility, >94.0%; MIC_90_, 2 mg/L) and colistin (susceptibility, >78.7%; MIC_90_, ≤8 mg/L) were active against all the *bla*_OXA-48-like_ isolates. These data are in agreement with those of the previous INFORM study where tigecycline (susceptibility, ≥92.5%; MIC_90_, 2 mg/L) and colistin (susceptibility, ≥78.7%; MIC_90_, ≤4 mg/L) were the only comparator agents active against all the *bla*_OXA-48-like_ isolates (25).

In a clinical study by Caston et al., treatment with CAZ-AVI was shown to have a significantly higher clinical cure rate at 14 days than that with comparator agents (85.7% vs 34.8%) in patients infected with carbapenemase-producing Enterobacterales, where majority of the infections were associated with OXA-48 producers (30). Furthermore, for infections caused by OXA-48-producing Enterobacterales, salvage therapy with CAZ-AVI has been shown to have a 61.5% clinical cure rate (31). Colistin and tigecycline monotherapies and combination therapies have been used to treat infections caused by OXA-48-producing Enterobacterales (31). The 2023 Infectious Diseases Society of America (IDSA) guidance on treatment of resistant Gram-negative infections recommends CAZ-AVI as the preferred treatment option for OXA-48-like-producing infections other than those of the urinary tract (32). Furthermore, the IDSA recommends tigecycline as an alternative treatment of infections caused by OXA-48-like-producing Enterobacterales excluding bloodstream infections and urinary tract infections (32). On the other hand, colistin is not suggested for treatment of infections caused by CRE due to increased mortality and nephrotoxicity associated with polymyxin-based regimens (32).

This study had limitations. First, as a pre-defined number of isolates were collected from each site, the results of this study cannot be interpreted as prevalence or used for epidemiological data. Second, there was a variation in the number of participating centers between years as well as the distribution of centers in each region. Third, the β-lactamase screening criteria were altered in 2015 to exclude the characterization of isolates that tested as imipenem or doripenem non-susceptible but only meropenem-susceptible (25). Hence, some isolates that were non-susceptible to the other carbapenemases could have been excluded from the screening. Although the majority of *bla*_OXA-48-like_ isolates co-carried an ESBL and isolates resistant to ceftazidime (MIC >8  µg/mL) were also screened for β-lactamase genes, some meropenem-susceptible isolates that did not co-carry ceftazidime-hydrolyzing β-lactamases could have been omitted from this analysis.

In this study, CAZ-AVI was highly active against all MBL-negative, *bla*_OXA-48-like_ Enterobacterales isolates collected from 2016 to 2020 with sustained activity from the previous time period (2012–2015). However, the overall increase in the geographic spread of *bla*_OXA-48-like_ Enterobacterales and those co-carrying MBLs is concerning, especially with currently available limited treatment options. Considering the limited treatment options against OXA-48-like Enterobacterales and the challenges of toxicity and increasing resistance with tigecycline and colistin, CAZ-AVI could be considered as a potential treatment option. Routine surveillance of these clinically relevant isolates and appropriate stewardship strategies may help identify emerging resistance mechanisms and effective treatment of infections.

## MATERIALS AND METHODS

Non-duplicate, clinically significant isolates (single isolate per patient) of Enterobacterales independent of age, sex, previous antimicrobial use, or medical history were collected in different sites across regions worldwide (AfME, APAC, Europe, LATAM, and North America) from patients between 2016 and 2020. Each site collected a pre-defined number of Enterobacterales isolates that were shipped to a central reference laboratory for (International Health Management Associates, Inc. Schaumburg, IL, USA) species conformation and antimicrobial susceptibility. Species confirmation was done using matrix-assisted laser desorption ionization-time of flight spectrometry (Bruker Biotyper MALDI-TOF, Bruker Daltonics, Billerica, MA, USA).

Susceptibility testing was performed by broth microdilution as per Clinical and Laboratory Standards Institute (CLSI) guidelines (33). Minimum-inhibitory concentrations (MICs) of all Enterobacterales were determined for CAZ-AVI, and a panel of comparator antimicrobial agents: amikacin, aztreonam, cefepime, colistin, imipenem, meropenem, piperacillin–tazobactam (Pip/Taz), and tigecycline and interpreted according to CLSI guidelines except tigecycline for which Food and Drug Administration (FDA)-approved breakpoints were used, and colistin, for which the version 12.0 of the European Committee on Antimicrobial Susceptibility Testing (EUCAST) breakpoint tables (34) was used. For MIC testing of CAZ-AVI, avibactam was fixed at a concentration of 4 mg/L in combination with doubling dilutions of ceftazidime (range, 0.015 mg/L to 256 mg/L).

All Enterobacteriaceae isolates that tested as nonsusceptible to meropenem (MICs, >1 mg/L), as well as *E. coli*, *K. pneumoniae*, *K. oxytoca*, *K. variicola,* and *P. mirabilis* isolates testing with ceftazidime and/or aztreonam (MICs, ≥2 mg/L) were screened for the presence of genes encoding *bla*_OXA-48-like_ and other β-lactamases (*bla*_KPC_, *bla*_NDM_*, bla*_IMP_*, bla*_VIM_*, bla*_SPM_*, bla*_GIM_*, bla*_TEM_*, bla*_SHV_*, bla*_CTX-M_*, bla*_VEB_*, bla*_PER_*, bla*_GES_*, bla*_ACC_*, bla*_ACT_*, bla*_CMY_*, bla*_DHA_*, bla_FOX_, bla*_MIR_*,* and *bla*_MOX_) using multiplex PCR, followed by amplification and sequencing of the full-length genes and comparison to publicly available databases as described previously (25). Isolates were considered genotypically MBL-positive if they had at least one of the genes: *bla*_IMP_, *bla*_VIM_, and *bla*_NDM_. Isolates that did not have any of the three genes were considered MBL-negative.

## SUPPLEMENTAL MATERIAL

Supplemental tables spectrum.01473-23-s0001.pdf Tables S1 to S5.

ASM does not own the copyrights to Supplemental Material that may be linked to, or accessed through, an article. The authors have granted ASM a non-exclusive, world-wide license to publish the Supplemental Material files. Please contact the corresponding author directly for reuse.
