# Supplementary material for: In vitro activity of ceftazidime–avibactam and comparators against OXA-48-like Enterobacterales collected between 2016 and 2020
Source: Microbiol Spectr. 2024 Feb 8;12(3):e01473-23. doi: 10.1128/spectrum.01473-23 (PMC10913439; doi:10.1128/spectrum.01473-23)
Supplement: Supplemental tables — Tables S1 to S5. [file spectrum.01473-23-s0001.pdf]

In vitro activity of ceftazidime-avibactam and comparators against OXA-48-Like Enterobacterales collected between 2016–2020

Gregory Stone[a], Mark Wise[b], and Eric Utt[a#]

Supplemantary Tables

Supplementary Table 1. Number of isolates of Enterobacterales collected and the number of sites from each country contributing these isolates between 2016-2020

Region	Country	No of Sites[1]	no of isolates
**AfME**		**25**	**8179**
	Cameroon	1	114
	Côte d Ivoire	1	71
	Israel	5	2057
	Jordan	1	124
	Kuwait	3	1634
	Morocco	3	785
	Nigeria	3	875
	Qatar	1	173
	Saudi Arabia	1	254
	South Africa	6	2092
**APAC**		**73**	**19284**
	Australia	5	2178
	China	24	4378
	Hong Kong	2	374
	India	10	2622
	Japan	6	1135
	Malaysia	3	491
	New Zealand	2	223
	Philippines	6	1799
	Singapore	1	88
	South Korea	5	2168
	Taiwan	5	1882
	Thailand	4	1946
**Europe**		**138**	**42069**
	Austria	2	293
	Belgium	7	2845
	Croatia	4	1056
	Czech Republic	4	2027
	Denmark	2	546
	Finland	1	157

	France	12	4246
	Germany	14	3873
	Greece	5	1900
	Hungary	5	1855
	Ireland	3	608
	Italy	13	3719
	Latvia	1	280
	Lithuania	2	533
	Netherlands	3	1034
	Poland	5	1634
	Portugal	5	2281
	Romania	4	884
	Russia	11	2377
	Slovenia	1	98
	Spain	12	4333
	Sweden	1	391
	Switzerland	2	533
	Turkey	7	1854
	Ukraine	3	534
	United Kingdom	9	2178
**LATAM**		**46**	**14142**
	Argentina	4	1807
	Brazil	14	2790
	Chile	3	1500
	Colombia	7	1874
	Costa Rica	1	269
	Dominican Republic	1	281
	Guatemala	2	624
	Mexico	7	3040
	Panama	3	493
	Venezuela	4	1464
**North America**			**10378**
	Canada	8	2024
	United States	30	8354

[1]indicates number of unique sites that collected isolates; AfME, Africa and Middle East; APAC, Asia Pacific; LATAM, Latin America.

Supplementary Table 2. Distribution of *bla*$_{OXA-48-like}$ Enterobacterales isolates collected globally and across different regions in 2016-2020

	**Global**	**AfME**	**APAC**	**Europe**	**LATAM**	**North America**
All Enterobacterales (N)	94052	8179	19284	42069	14142	10378
All OXA-48-Like [n (% of N)]	1690 (1.8%)	220 (2.7%)	598 (3.1%)	812 (1.9%)	49 (0.3%)	11 (0.1%)
OXA-48-Like, ESBL (-), MBL (-) [n (% of all OXA-48-like)]	150 (8.9%)	29 (13.2%)	16 (2.7%)	99 (12.2%)	5 (10.2%)	1 (9.1%)
OXA-48-Like, ESBL (+), MBL (-) [n (% of all OXA-48-like)]	1190 (70.4%)	172 (78.2%)	308 (51.5%)	666 (82.0%)	40 (81.6%)	4 (36.4%)
OXA-48-Like, ESBL (-) MBL (+) [n (% of all OXA-48-like)]	38 (2.2%)	4 (1.8%)	24 (4.0%)	7 (0.9%)	1 (2.0%)	2 (18.2%)
OXA-48-Like, ESBL (+) MBL (+) [n (% of all OXA-48-like)]	312 (18.5%)	15 (6.8%)	250 (41.8%)	40 (4.9%)	3 (6.1%)	4 (36.4%)

AfME, Africa and Middle East; APAC, Asia Pacific; ESBL, extended spectrum β-lactamase; LATAM, Latin America; MBL, metallo-β-lactamase; N, total number of isolates; n, number of *bla*$_{OXA-48-like}$ isolates.

Supplementary Table 3. Distribution of variants of $\textit{bla}_{\text{OXA-48-like}}$ Enterobacterales isolates collected globally and across different regions in 2016-2020

	**Global (N=1690)**	**AfME (N=220)**	**APAC (N=598)**	**Europe (N=812)**	**LATAM (N=49)**	**North America (N=11)**
OXA-48 [n (% of N)]	848 (50.2%)	88 (40.0%)	20 (3.3%)	724 (89.2%)	10 (20.4%)	6 (54.5%)
OXA-232 [n (% of N)]	496 (29.3%)	32 (14.5%)	392 (65.6%)	51 (6.3%)	19 (38.8%)	2 (18.2%)
OXA-181 [n (% of N)]	304 (18.0%)	100 (45.5%)	182 (30.4%)	16 (2.0%)	3 (6.1%)	3 (27.3%)
Others[a] [n (% of N)]	42 (2.5%)	0 (0.0%)	4 (0.7%)	33 (4.1%)	17 (34.7%)	0 (0.0%)

[a]Other variants include OXA-244 (n=17, n=1 in APAC, and n=16 in Europe), OXA-163 (n=12 in LATAM), OXA-162 (n=5 in Europe), OXA-370 (n=5 in LATAM), OXA-484 (n=1 in APAC), OXA-48-TYPE (n=1 in APAC), and OXA-48-New Variant (n=1 in APAC).
N, total number of isolates; n, number of $\textit{bla}_{\text{OXA-48-like}}$ isolates; AfME, Africa and Middle East; APAC, Asia Pacific; LATAM, Latin America.

Supplementary Table 4. Antimicrobial activity of CAZ-AVI and comparators against *bla*$_{OXA-48-like}$, MBL-positive Enterobacterales isolates collected globally in 2016-2020.

	**%S**	**%R**	**MIC$_{50}$**	**MIC$_{90}$**	**MIC range**
**OXA-48-Like, MBL (+), ESBL (-) (N=38)**					
Amikacin	15.8	78.9	>64	>64	1->64
Aztreonam	65.8	28.9	0.5	32	0.03->64
CAZ-AVI	0.0	100.0	>128	>128	64->128
Cefepime	2.6	97.4	>32	>32	1->32
Colistin[1]	78.9	21.1	0.5	>8	0.12->8
Imipenem	0.0	100.0	>8	>8	8->8
Meropenem	0.0	100.0	>16	>16	8->16
Pip/Taz	0.0	100.0	>64	>128	64->128
Tigecycline[2]	86.8	2.6	0.5	4	0.06-8
**OXA-48-Like, ESBL (+), MBL (+) (N=312)**					
Amikacin	18.9	77.9	>64	>64	0.25->64
Aztreonam	0.3	99.4	>64	>128	1->128
CAZ-AVI	0.3	99.7	64	>128	1->128
Cefepime	0.0	99.4	>32	>32	4->32
Colistin[1]	81.7	18.3	0.5	>8	0.06->8
Imipenem	0.9	97.6	>8	>8	0.06->8
Meropenem	0.6	97.8	>16	>16	0.5->16
Pip/Taz	0.6	99.4	>64	>128	2->128
Tigecycline[2]	95.5	0.0	1	2	0.06-4

[1]EUCAST breakpoints have been used; [2]FDA approved breakpoints have been used; N, total number of isolates; S, susceptibility; R, resistance; CAZ-AVI, ceftazidime avibactam; Pip/taz, piperacillin/tazobactam; MIC, minimum inhibitory concentration; MIC$_{50}$ minimum inhibitory concentration required to inhibit 50% of the organisms; MIC$_{90}$ minimum inhibitory concentration required to inhibit 90% of the organisms; ESBL, extended spectrum β-lactamase; MBL, metallo-β-lactamase.

Supplementary Table 5. Distribution of ESBLs and MBLs among variants of $\textit{bla}_{\text{OXA-48-like}}$ Enterobacterales isolates collected globally and across different regions in 2016-2020.

	**OXA-48**	**OXA-181**	**OXA-232**
**Global**			
All (N)	848	304	496
ESBL (-), MBL (-) [n (% of N)]	111 (13.1%)	22 (7.2%)	13 (2.6%)
ESBL (+), MBL (-) [n (% of N)]	672 (79.2%)	162 (53.3%)	320 (64.5%)
MBL (+), ESBL (±) [n (% of N)]	65 (7.7%)	120 (39.5%)	163 (32.9%)
**AfME**			
All (N)	88	100	32
ESBL (-), MBL (-) [n (% of N)]	15 (17.0%)	11 (11.0%)	3 (9.4%)
ESBL (+), MBL (-) [n (% of N)]	59 (67.0%)	87 (87.0%)	26 (81.3%)
MBL (+), ESBL (±) [n (% of N)]	14 (15.9%)	2 (2.0%)	3 (9.4%)
**APAC**			
All (N)	20	182	392
ESBL (-), MBL (-) [n (% of N)]	3 (15.0%)	7 (3.8%)	4 (1.0%)
ESBL (+), MBL (-) [n (% of N)]	7 (35.0%)	61 (33.5%)	238 (60.7%)
MBL (+), ESBL (±) [n (% of N)]	10 (50.0%)	114 (62.6%)	150 (38.3%)
**Europe**			
All (N)	724	16	51
ESBL (-), MBL (-) [n (% of N)]	92 (12.7%)	1 (6.3%)	4 (7.8%)
ESBL (+), MBL (-) [n (% of N)]	596 (82.3%)	12 (75.0%)	40 (78.4%)
MBL (+), ESBL (±) [n (% of N)]	36 (5.0%)	3 (18.8%)	7 (13.7%)
**LATAM**			
All (N)	10	3	19
ESBL (-), MBL (-) [n (% of N)]	1 (10.0%)	2 (66.7%)	1 (5.3%)
ESBL (+), MBL (-) [n (% of N)]	7 (70.0%)	1 (33.3%)	16 (84.2%)
MBL (+), ESBL (±) [n (% of N)]	2 (20.0%)	0 (0.0%)	1 (5.3%)

North America			
All (N)	6	3	2
ESBL (-), MBL (-) [n (% of N)]	0 (0.0%)	1 (33.3%)	0 (0.0%)
ESBL (+), MBL (-) [n (% of N)]	3 (50.0%)	1 (33.3%)	0 (0.0%)
MBL (+), ESBL (±) [n (% of N)]	3 (50.0%)	1 (33.3%)	2 (100.0%)

N, total number of isolates of each OXA-48-like variant; n, number of isolates that are MBL/ESBL ±; AfME, Africa and Middle East; APAC, Asia Pacific; LATAM, Latin America; ESBL, extended spectrum β-lactamase; MBL, metallo-β-lactamase.
